# Supplementary material for: Myostatin and Follistatin—New Kids on the Block in the Diagnosis of Sarcopenia in IBD and Possible Therapeutic Implications
Source: Biomedicines. 2021 Sep 23;9(10):1301. doi: 10.3390/biomedicines9101301 (PMC8533148; doi:10.3390/biomedicines9101301)
Supplement: Supplementary file 1 [file biomedicines-09-01301-s001.zip › biomedicines-1354876-supplementary.pdf]

Table S1. Genetic factors of sarcopenia associated with IBD.

	Location	Sarcopenia study, phenotype	Marker (LOD), candidate gene, variant	IBD study, phenotype	Marker (LOD score), candidate gene, variant
**Whole genome linkage studies**	1p36.13<br>1p36 (IBD)	Livshits et al., 2007 (50), total body lean mass	D1S199 (2.17)	Cho et al., 2000 (51), OMIM 605225, IBD7	D1S157, D1S2697, D1S3669 (2.65)
	7p15.3-15.1<br>7p15.3 (IBD)	Livshits et al., 2007 (50), total body lean mass	D7S629, D7S516 (2.86), *PPP1R17*, *GARS*	OMIM 266600, Crohn's disease-associated growth failure	*IL6*
	7q21<br>7q21.12 (IBD)	Wang et al. 2008 (52), total body lean mass and hip BMD;<br>Deng et al. 2007 (53), total body lean mass and femoral neck cross-sectional area	GATA24D12P (2.52), *HGF*; GATA3F01 (4.65)	Cho et al., 2000 (51), OMIM 612244, IBD13	D7S669 (3.08), *ABCB1*
	7q32	Wang et al. 2008 (52),	MFD442-	Dideberg et al., 2007 (55);	*IRF5*

	7q32.1 (IBD)	total body lean mass and hip BMD<br>Zhao et al. 2007 (54); lean mass	GTTT002 (2.67), *LEP*;<br>GATA104 (2.79)	OMIM 612245, IBD14	
	10q24<br>10q23-q24 (IBD)	Deng et al. 2007 (53), total body lean mass and femoral neck buckling ratio	GATA64A09 (3.59)<br>*CYP17A1*	Hampe et al., 1999 (56); OMIM 612288,<br>IBD20	D10S547 and D10S20 (2.3),<br>*DLG5*
	12p12.3-12p13.2<br>12q23.2<br>12p13.2-q24.1 (IBD)	Karasik et al. 2009 (57), lower limb<br>muscle strength and shaft cross section area	*TNFRSF1A* (3.49)	Satsangi et al. 1996 (58); OMIM 601458,<br>IBD2	D12S83 (5.47)
		Chagnon et al. 2001(59), fat-free mass	*IGF1*		
	18p11.31<br>18p11 (IBD)	De Mars et al. (2008)(60),<br>Isometric knee extension torque	rs341173 (2.39)	Parkes et al. 2007 (61),<br>OMIM 612354, IBD21	rs2542151, *PTPN2*

**Gene association studies**	*VDR* gene	Pratt et al. 2019 (31) association to muscle strength or/and physical function or/and body composition in 9 studies	rs1544410 (BsmI) - 3 studies, rs2228570 (FokI) - 4 studies, rs7975232 (ApaI) - 1 study, rs7136534 - 1 study	Pei et al. 2011 (62); UC susceptibility in Han Chinese<br><br>Naderi et al. 2008 (63)**;** CD susceptibility in Irian patients	rs1544410 (BsmI)<br><br>rs2228570 (FokI)
	NR3C1 gene	van Rossum et al. 2004 (64), total body lean mass, arm pull strength and high jump	rs6189/rs6190 (p.E22R/E23K)	Krupoves et al., 2011 (65), GCs resistance in paediatric CD Skrzypczak-Zielinska et al., 2021 (66), glucocorticoid resistance in UC patients	rs6189/rs6190 (p.E22R/E23K)<br><br>rs56149945 (c.1088A>G p.N363S)

				De Iudicibus et al. 2007 (67), increased sensitivity in GC responders versus GC dependents in IBD<br>De Iudicibus et al. 2011 (68), association with steroid response versus dependency in paediatric IBD patients	rs41423247<br>(1184+646C>G, BclI)
	IL6 gene	Pereira, et al. 2013 (69), physical function<br>Dedoussis et al. 2004 (70), muscle mass in school boys<br>Roth et al. 2003 (71), total body lean mass in adults	rs1800795<br>(c.-84-153C>G)	Liu et al. 2020 (72), risk of IBD both in overall and Caucasian population<br><br>Bek et al. 2016 (73), Bank et al. 2014 (74), anti-TNF treatment response in IBD	rs1800795<br>(c.-84-153C>G)<br><br>rs10499563
	IL10 gene	Pereira, et al. 2013 (69), physical function	rs1800896<br>(c.-15+128A>G)	Quiroz-Cruz et al. 2020 (75), increased risk for both UC and	rs1800896<br>(c.-15+128A>G)

				CD<br>risk for early onset of UC and use of steroid treatment<br><br>Lin et al. 2017 (76), paediatric IBD susceptibility	rs3024505<br><br>rs304496<br>(c.61+25786T>A/G/C)
	TNF gene	Tiainen, et al. 2012 (77), physical function<br><br>Li, et al. 2016 (78), muscle strength<br><br>Pereira, et al. 2013 (69), physical function<br><br>Liu et al. 2008 (79), muscle mass in men	rs361525<br><br>rs1799964<br><br>rs1800629<br><br>rs1800630<br>rs1799964	Bank et al. 2014 (74), anti-TNF treatment response in IBD<br><br>Senhaji et al. 2016 (80), IBD susceptibility in Moroccan patients	rs361525<br><br>rs1800629

	LTA gene	Li, et al. 2016 (78), muscle strength in women<br><br>muscle strength in men	rs1800629, rs909253, rs1041981<br><br>rs2239704	Taylor et al. 2001 (44), response to anti-TNF treatment in CD	NcoITNFc-aa13L-aa26 (haplotype)
**Gene expression, miRNA studies**	mi-206	Kim et al. 2006 (81), promoting myoblast differentiation during myogenesis	*GJA1*, *FSTL1*, *POLA1*, *UTRN*	Wu et al. 2017 (82), miR-126 overexpression enhanced UC inflammatory activity by downregulating the expression of Iκ-B*α*	NF-κB signalling pathway
	mi-146a	Kuang et al. 2009 (83), muscle differentiation and proliferation by inhibiting *NUMB* gene expression	*NUMB*	Szűcs et al., 2016 (84); anti-inflammatory TGF-β plays an important role in the regulation of the expression of miR-146a, NF-κB, IL-1β and the TNF-*α* pathway	NF-κB, IL-1β and the TNF-*α* pathway

LOD - logarithm (base 10) of odds, *PPP1R17* - protein phosphatase 1 regulatory subunit 17, *GARS* - glycyl-tRNA synthetase 1, IL6 – interleukin 6, ABCB1- ATP binding cassette subfamily B member 1, BMD - bone mineral density, *HGF* - hepatocyte growth factor, LEP – leptin, *IRF5* - interferon regulatory factor 5, *CYP17A1* - cytochrome P450 family 17 subfamily A member 1, *DLG5* - discs large MAGUK scaffold protein 5, *TNFRSF1A* - TNF receptor superfamily member 1A, *IGF1* - insulin like growth factor 1, *PTPN2* - protein tyrosine phosphatase non-receptor type 2, GC – glucocorticoid, *VDR* - vitamin D receptor, *NR3C1* -nuclear receptor subfamily 3 group C member Il6 - interleukin 6, *IL10* - interleukin 10, *TNF* - tumour necrosis factor, *LTA* - lymphotoxin alpha , *GJA1* - gap junction protein alpha, *FSTL1* - follistatin like 1, *POLA1* - DNA polymerase alpha 1, *UTRN* - utrophin, *NUMB* - NUMB endocytic adaptor protein, NF-κB - nuclear factor kappa B subunit, Iκ-Bα - NFKB inhibitor alpha, IL-1β – interleukin 1 beta,
